# A Genome-Wide Perspective of miRNAome in Response to High Temperature, Salinity and Drought Stresses in *Brassica juncea* (Czern) L

**DOI:** 10.1371/journal.pone.0092456

**Published:** 2014-03-26

**Authors:** Ankur R. Bhardwaj, Gopal Joshi, Ritu Pandey, Bharti Kukreja, Shailendra Goel, Arun Jagannath, Amar Kumar, Surekha Katiyar-Agarwal, Manu Agarwal

**Affiliations:** 1 Department of Botany, University of Delhi, Delhi, India; 2 Department of Plant Molecular Biology, University of Delhi, South Campus, Delhi, India; Cankiri Karatekin University, Turkey

## Abstract

Micro RNAs (miRNAs) are involved in diverse biological processes including adaptive response towards abiotic stresses. To unravel small RNAs and more specifically miRNAs that can potentially regulate determinants of abiotic stress tolerance, next generation sequencing of *B. juncea* seedlings subjected to high temperature, high salt and drought conditions was carried out. With the help of UEA sRNA workbench software package, 51 conserved miRNAs belonging to 30 miRNA families were identified. As there was limited genomic information available for *B. juncea*, we generated and assembled its genome sequence at a low coverage. Using the generated sequence and other publically available *Brassica* genomic/transcriptomic resources as mapping reference, 126 novel (not reported in any plant species) were discovered for the first time in *B. juncea*. Further analysis also revealed existence of 32 and 37 star sequences for conserved and novel miRNAs, respectively. The expression of selected conserved and novel miRNAs under conditions of different abiotic stresses was revalidated through universal TaqMan based real time PCR. Putative targets of identified conserved and novel miRNAs were predicted in *B. rapa* to gain insights into functional roles manifested by *B. juncea* miRNAs. Furthermore, SPL2-like, ARF17-like and a NAC domain containing protein were experimentally validated as targets of miR156, miR160 and miR164 respectively. Investigation of gene ontologies linked with targets of known and novel miRNAs forecasted their involvement in various biological functions.

## Introduction

Crop yield is determined by a multitude of factors that includes genetic makeup and both biological and non-biological challenges. Abiotic stresses for e.g., exposure to short/long periods of non-optimal temperatures, physical/physiological non-availability of water and soil salinity/sodicity are the major factors affecting the yield. Additionally, in field conditions these stresses occur in combination or succession further limiting the yield potential. For example, high temperature in combination with soil salinity or drought results in heavy yield loss [1], [2]. At the molecular level the immediate effect of stress injury is induction/activation of specific genes or proteins that prevent cellular damage and also aid in recovery [3], [4]. Genome wide expression analysis has revealed the existence of a complex array of stress specific adaptive response that is mediated by induced/activated genes/proteins. Though there are specific pathways for each stress, some key proteins act as nodal components wherein stress responsive signal transduction pathways integrate [5], [6].

Small RNAs have emerged as ubiquitous key molecules regulating gene expression [7]–[10]. The repressive effects of small RNAs have been observed at transcriptional, post-transcriptional as well as translational levels [11]. In plants, small RNAs and more specifically, miRNAs have been functionally associated with development [12], [13], biotic [14]–[17] as well as abiotic stresses [18], [19]. Following the first report of miRNA discovery in plants [7] significant advancements and refinements have been made in sequencing technologies which has led to discovery of small RNAs at an unprecedented scale. The first high-throughput sequencing approach to discover small RNAs in plants was adopted by Lu *et al.* (2005) [20] and since then 7389 miRNAs have been discovered in 73 different plant species (miRBase v20).

The involvement of miRNAs in controlling cellular response to abiotic stresses has been well documented. Regulation of miRNAs by abiotic stresses was initially reported independently by Jones-Rhoades *et al.* [21] and Sunkar *et al.* in 2004 [22]. Subsequently, a number of reports were published which reiterated that miRNAs are themselves regulated by abiotic factors and they, in turn, control the levels of target genes involved in governing the stress responses. Two of the most glaring examples are miR398 and miR395, which have been repeatedly shown by independent groups to regulate cellular response in many different stresses [23]–[28]. Other miRNAs that play significant role during abiotic stresses include miR169 family members that regulate drought stress response in *Arabidopsis*, miR399 governing targeted protein degradation and phosphate distribution, and miR393 helping plants to survive limited nitrogen condition [29]–[34]. Recently, Song *et al*. has shown the involvement of miR394 in salinity and drought stress via regulation of *LEAF CURLING RESPONSIVENESS* (*LCR*) which encodes a F-box containing protein. It was demonstrated that MIR394a overexpression and *lcr* mutant lines in Arabidopsis were hypersensitive to salinity. Moreover, constitutive overexpression of *LCR* exhibited enhanced tolerance against drought while plants overexpressing miR394-resistant *LCR* were drought susceptible [35].

Initial discovery of miRNA in *Brassica* was reported in 2007 when Xie *et al.* identified 21 miRNAs in *B. napus* using computational methods [36]. They also demonstrated differential modulation of five miRNAs in response to auxin, cadmium stress and sulphate deprivation. Pant *et al.* (2009) for the first time performed deep sequencing of small RNA libraries to identify phosphate deprivation responsive miRNAs in *B. rapa* and *Arabidopsis* [37]. A comprehensive study on global miRNA response against phosphate deficiency and cadmium stress in *B. napus* was performed by Huang *et al.* [38]. They further validated *BnAPS3*, *BnAPS4* and *BnSultr2;1* as targets of miR395. Subsequently, several studies have reported both conserved and novel miRNAs from *B. rapa* [39]–[41], *B. napus* [42]–[45], *B. oleracea* [46] and *B. juncea* [47].

*Brassica juncea* (Czern) L. (AABB, 2n = 36) is an amphidiploid species which originated from interspecies crosses between *B. rapa* (AA, 2n = 20) and *B. nigra* (BB, 2n = 16). It is commonly known as Indian mustard or brown seeded mustard and widely grown as an oilseed crop [48], [49]. Like all crop systems, exposure of soil salinity, high temperature and drought at specific developmental stages of Brassicas leads to compromised growth and development [50]–[54]. For example, high temperature stress during pod development results in reduced seed setting [55]. Similar yield loss has been observed upon drought treatment during pod development [56]. Increased soil salinity during early development exhibit reduction in shoot and root length, decreased leaf relative water content and increased oxidative stress [57]. Hence, to discover the *B. juncea* stress-responsive miRNAs we utilized highthroughput sequencing to sequence millions of small RNA molecules in *B. juncea* seedlings subjected to supra-optimal temperatures, high salt concentrations and drought stress. Present study provides a genome-wide perspective of miRNAome under three abiotic stresses that are pertinent with respect to their detrimental effects on crop productivity. The discovered miRNAs and their targets have a potential to be used either to study the molecular mechanisms of stress signaling or for manipulating the tolerance levels against abiotic stresses.

## Materials and Methods

### Plant Material and Growth Conditions

*Brassica juncea* var. varuna seeds were obtained from Indian Agricultural Research Institute (IARI), Delhi, India. Seeds were surface sterilized with 2% sodium hypochlorite solution for ten minutes, followed by extensive washes with sterile water and hydroponically grown in a plastic container lined at the top with muslin cloth. The trays were placed in a growth room (Bhanu Biotech, India) maintained at 24°C±1 with a 16 h day/8 h night photoperiod.

### Stress Conditions and Treatment

Seven day old seedlings were subjected to various abiotic stresses. Heat stress was given at two different temperatures, 35°C and 42°C, for 30 min, 2 h, 4 h and 8 h by decanting the water from the growth container and placing it in a BOD incubator (Scientific Systems, India). Salinity stress was given at two different concentrations, 150 mM NaCl and 250 mM NaCl, for 3 h, 6 h, 12 h and 24 h by replacing the water from the container with NaCl solution. Similarly, drought stress was given with 20% PEG and 300 mM mannitol for 1 h, 3 h, 6 h and 12 h. Untreated seedlings were taken as control. Equal amounts of seedling tissues obtained from all time-points of each stress were pooled as one sample. In this manner, three separate samples were collected for three different abiotic stresses, which are hereafter referred as BJH (*B. juncea* high temperature stress), BJS (*B. juncea* salinity stress) and BJD (*B. juncea* drought stress). Untreated seedlings were named as BJC (*B. juncea* control). Tissues were snap-frozen in liquid nitrogen and processed for RNA extraction.

### Small RNA Library Construction and Sequencing

Enriched low molecular weight (LMW) RNA was isolated from BJC, BJH, BJS and BJD samples using GITC-LiCl method with some modifications [58]. Tissue was homogenized in liquid nitrogen and total RNA was extracted using GITC buffer and phenol-chloroform purification steps followed by ethanol precipitation. Total RNA was re-precipitated with lithium chloride to extract low molecular weight RNA. Extracted enriched-LMW RNA was quantified using spectrophotometer (Biorad, USA) and its integrity was checked by 15% Urea-PAGE. Four small RNA libraries *B. juncea* control (BJC), *B. juncea* high temperature stressed (BJH), *B. juncea* salinity stressed (BJS) and *B. juncea* drought stressed (BJD), were prepared utilizing Illumina small RNA sample preparation kit v1 according to manufacturer’s instructions. Briefly, equal amount of enriched-LMW RNA (50 μg) was size fractionated on 15% TBE-urea gel and small RNA fraction (18–40 nt) was excised and purified. RNA adapters were ligated on both ends using T4 RNA ligase followed by cDNA preparation utilizing Superscript II (Invitrogen, USA). The prepared cDNA was PCR amplified and the amplicon of approximately 100 bp was excised from a 6% TBE gel. The quality and quantity of prepared libraries was evaluated utilizing DNA 1000 kit on a bioanalyzer (Agilent, USA). Ultra-deep parallel sequencing was performed using Illumina Genome Analyzer IIx at University of Delhi South Campus, Delhi, India.

### Genomic DNA Library Preparation and Sequencing

Genomic DNA was isolated from *B. juncea* seedlings using CTAB method [59]. The quality and quantity of the extracted DNA was assessed by electrophoresis on a 0.8% agarose gel and spectrophotometer (Biorad, USA), respectively. Three μg gDNA was used to prepare a genomic DNA library utilizing Illumina DNA sample preparation kit according to manufacturer’s instructions. High-throughput pair-end sequencing (2×75 cycles) was performed on Illumina GA IIx sequencer at University of Delhi South Campus, India. High quality reads were assembled through Velvet/Oasis assemblers [60], [61].

### Data Analysis

Images obtained from Illumina GA IIx were converted into text files using genome analyzer pipeline software provided with the GA IIx. The UEA small RNA workbench v2.4 was then used for further analysis [62]. Briefly, after trimming of adapter sequences, tags of length ranging from 16–30 nt were retained and all non-small RNA sequences like low complexity sequences (simple sequence repeats) and degradation species (fragments of tRNA, rRNA, snRNA, snoRNA) were removed. The putative small RNA reads thus obtained were screened for the presence of conserved miRNAs based on their perfect homology with miRBase version 20 [63]–[65]. The remaining dataset was mapped onto the *B. juncea* genome reference dataset (generated in our lab by sequencing on Illumina plateform at 8× coverage; manuscript under preparation), *B. rapa* reference dataset (obtained from BrassicaDB) and reference dataset of other Brassicas (obtained from NCBI). The flanking sequences of varying length on both sides of the mapping position were extracted and processed for prediction of secondary hairpin structure. The secondary structures conforming to the screening criteria inbuilt in the UEA server were used to find out conserved (miRNAs that matched perfectly with sequences of miRBase v20) and novel miRNAs (miRNAs with ≥3 mismatches with sequences reported in miRBase v20). Small RNA sequencing data has been submitted at NCBI in Gene Expression Omnibus database under the accession GES53242.

### Target Transcript Analysis and Validation

Targets of both the conserved and novel miRNAs were predicted utilizing psRNA Target server [66] with no more than three penalty score. Cleavage sites of target mRNAs for few conserved miRNAs were experimentally mapped by RLM-RACE. Briefly, poly A^+^ RNA was isolated by using PolyATract mRNA isolation system IV (Promega, USA). PolyA^+^ mRNA (25 ng) was ligated to RNA adapter provided in First Choice RLM-RACE kit (Ambion, USA) using T4 RNA ligase. Nested gene-specific primers were utilized for amplification of the target gene. The amplicon was gel purified, cloned in pGEMT Easy vector (Promega, USA) and multiple clones were sequenced to map the cleavage site.

### Quantitative Real Time PCR of miRNAs and Target Genes

Ten microgram of RNA was treated with 2 U of RNase free DNase I (NEB, USA) followed by phenol-chloroform extraction and precipitation. Two μg of DNase treated RNA was polyadenylated using Poly A-tailing kit (Ambion, USA) as per manufacturer’s instructions and column purified with RNAeasy minelute kit (Qiagen, GmBH). Subsequently, each sample was reverse transcribed using 1 μg of linker primer and Superscript III (Invitrogen, USA). Quantitative real time PCR of miRNA sequences was performed on Realplex^2^ mastercycler (Eppendorf, Germany). miRNA validation and profiling was done with miRNA specific forward primer (800 nM), universal reverse primer (800 nM), and a TaqMan probe (200 nM) that binds in the linker primer region, using FastStart Universal Probe Master chemistry (Roche, USA). 5S rRNA sequence was used as endogenous control. In case of target genes DNase free RNA was reverse transcribed using iScript reverse transcription kit (Biorad, USA). Target gene expression profiling was done using gene-specific primers and KAPA SYBR FAST chemistry (Kapa biosystems, USA). Actin was used as an internal reference control. C_T_ values obtained through qPCR were analyzed using delta delta C_T_ method to calculate relative fold change values. The details of the primers used in qPCR and other experiments are given in supplementary table S1.

## Results

Four small RNA libraries were prepared from hydroponically grown 7-d old *B. juncea* seedlings. Highthroughput sequencing of these libraries resulted in more than 25 million short fragments. Following removal of redundancy nearly 12.6 million sequences were found to be unique. We used UEA sRNA workbench v2.4 for identification of conserved and prediction of novel miRNAs [62].

### Analysis of Small RNA Libraries

Sequencing reads obtained from each of the four libraries were pooled and downstream analysis was carried out on this pooled dataset. Approximately 84% of purity filtered tags had 3′ adapter sequence indicating that most of these sequences were in the desired size range of 21–24 nucleotides (Supplementary Table S2). The adapter sequence was computationally trimmed and digital expression of each sequence was determined by counting the number of times it was represented in the parent library. The removal of redundancy resulted in a dataset of approximately 12.6 million unique reads. These unique sequences were processed through an elimination pipeline (filter module of UEA sRNA workbench) for size selecting the sequences (falling in 16–30 nucleotides range) and removing the degradation species (for e.g. fragments of tRNA, rRNA, sn/snoRNA), low complexity sequences (SSRs/TRs) and invalid tags (having N). The percentage of sequences that did not fall in the size range of 16–30 nt was 19% and 31.9% in case of redundant and unique sequences respectively (Table 1). The percentage of degradation species comprised upto 4.5% in redundant and 0.33% in unique tags indicating that these molecules were over-represented in the redundant dataset. However, the percentage representation of the low complexity sequences (0.006% in redundant and 0.009% in unique) and the invalid tags (1.0% in redundant and 1.5% in unique) was similar in both the redundant and the unique tags (Table 1). The dataset obtained after the elimination pipeline was considered as the putative small RNA dataset. The percentage of the putative small RNAs was 75.4% (18.9 million) and 66.2% (8.3 million) in the redundant and the unique datasets, respectively (Table 1). Majority of the small RNA population comprised of 24 nt long sequences followed by 23 and 21 nt conforming to previous reports that 24 nt long siRNAs contribute largely to the small RNAs in a cell [67]–[71]. The absolute number of small RNA transcripts with respect to length distribution is depicted in supplementary figure S1.

**Table 1:** Filtering of small RNA reads. Purity filtered sequences were processed through an elimination pipeline.

Category	Number and percentageof redundant reads	Number and percentageof unique reads
Total reads	25065874 (100%)	12642620 (100%)
Sequences length (<16nt and >30nt)	4771628 (19%)	4032111 (31.9%)
Low-complexity sequences	1522 (0.006)	1200 (0.009)
Invalid sequences (containing N)	255410 (1%)	196190 (1.6%)
tRNA/rRNA matches	1130945 (4.5%)	41752 (0.3%)
Remaining sRNAs (Putative sRNAs)	18906369 (75.4%)	8371367 (66.2%)

The number and percentage (in parentheses) of the sequences removed for redundant (column 2) and unique sequences (column 3) are tabulated. The category of the removed sequences is represented in column 1.

The putative small RNA population was mapped onto the available *Brassica* genomic and transcriptomic resources (*B. rapa* BACs obtained from *Brassica*DB and EST/GSS of other *Brassica* species obtained from NCBI). Because of the limited genomic resources we also performed deep sequencing of *B. juncea* genome at 8× coverage to generate a reference for mapping of putative small RNAs. Out of 8.3 million sequences, 28.6% of the sequences mapped on the *B. juncea* genome dataset generated in this study, 18% of the sequences mapped on the *B. rapa* genome dataset while 11.1% of the sequences mapped on the genomic dataset comprising of other *Brassica* species. To fish out authentic pre microRNAs, sequence falling within a varying window length on both sides of the mapped region was extracted and subjected to RNA folding module of UEA server.

### Identification of the Conserved and Novel miRNAs

Based on the initial survey of folded structure and homology of mature miRNAs sequences with miRBase v19, we were able to identify 51 conserved miRNAs representing 30 miRNA families. All these sequences were identical to at least one of the previously reported sequence in the miRBase. A list of the identified miRNA families and the number of members for each family that were identified in this study are presented in Table 2. The sequence of the most abundant member (based on read counts of control sample) is also presented in Table 2. The highest number of miRNA members belong to miR169 family (5 members) followed by 4 members each in miR156 and miR171 families. The sequence of the miRNA, their absolute number and fold change under stress conditions with respect to that of control sample is presented in supplementary table S3. Digital gene expression revealed that many conserved miRNAs responded significantly (atleast two fold up/down regulation) to one or more abiotic stresses. For example, 15 miRNAs showed ≥2 fold change under drought stress, same number (15) of miRNAs responded to high temperature stress whereas 13 miRNAs were modulated by salinity stress. Five of the miRNAs were regulated by both high temperature and salinity stress. Similarly, two miRNAs each were modulated by both high temperature and drought stress as well as by both salinity and drought stress. The number of miRNAs that were significantly regulated by one or multiple stress condition is provided in supplementary figure S2. The precursors of these miRNAs were derived from *B. juncea*, *B. rapa* and other *Brassica* species. Multiple precursors were identified for many conserved miRNAs (Supplementary Table S4). Star sequences for 32 of the 51 conserved miRNAs were also detected in the sequenced data. Additional details on the precursors and the star sequences are presented in supplementary table S4.

**Table 2:** Family wise distribution of conserved miRNAs in *B. juncea*.

miRFAM	Number of miRNA members	Sequence of the most abundant member
mir169	5	UAGCCAAGGAUGACUUGCCUG
mir156	4	UGACAGAAGAGAGUGAGCAC
mir171	4	UGAUUGAGCCGCGUCAAUAUC
mir172	3	AGAAUCUUGAUGAUGCUGCAU
mir2111	3	GUCCUCGGGAUGCGGAUUACC
mir395	3	CUGAAGUGUUUGGGGGAACUC
mir399	3	UGCCAAAGGAGAGUUGCCCUG
mir164	2	UGGAGAAGCAGGGCACGUGCA
mir167	2	UGAAGCUGCCAGCAUGAUCUA
mir319	2	UUGGACUGAAGGGAGCUCCCU
mir397	1	UCAUUGAGUGCAGCGUUGAUGU
mir157	1	UUGACAGAAGAUAGAGAGCAC
mir158	1	UCCCAAAUGUAGACAAAGCA
mir159	1	UUUGGAUUGAAGGGAGCUCUA
mir160	1	UGCCUGGCUCCCUGUAUGCCA
mir161	1	UCAAUGCACUGAAAGUGACUA
mir162	1	UCGAUAAACCUCUGCAUCCAG
mir165	1	UCGGACCAGGCUUCAUCCCCC
mir166	1	UCGGACCAGGCUUCAUUCCCC
mir168	1	UCGCUUGGUGCAGGUCGGGAA
mir390	1	AAGCUCAGGAGGGAUAGCGCC
mir391	1	UUCGCAGGAGAGAUAGCGCCA
mir393	1	UCCAAAGGGAUCGCAUUGAUCC
mir394	1	UUGGCAUUCUGUCCACCUCC
mir396	1	GCUCAAGAAAGCUGUGGGAAA
mir398	1	GGAGUGUCAUGAGAACACGGA
mir403	1	UUAGAUUCACGCACAAACUCG
mir828	1	UCUUGCUUAAAUGAGUAUUCCA
mir845	1	CGGCUCUGAUACCAAUUGAUG
mir858	1	UUCGUUGUCUGUUCGACCUUG

The number of miRNA members in each family with the sequence of the most abundant miRNA member is presented.

The remaining population of the putative small RNAs was further searched for variants of the reported miRNAs in miRBase. In this case we allowed a maximum of 2 base mismatches against previously reported miRNA sequences in miRBase. Variant miRNAs that had a cumulative read count of ≥20 were retained while others were discarded. The sequences of the variant miRNAs, their miRNA family and absolute counts in the sequenced libraries are presented in supplementary table S5. Nucleotide sequence homology of identified conserved miRNAs was studied in other members of *Brassica*ceae (*B. rapa*, *B. napus*, *B. oleracea*, *A. thaliana* and *A*. *lyrata*) (Table 3). It was observed that out of the 51 conserved miRNAs sequences identified in *B. juncea,* 27 miRNA sequences were identical to previously reported miRNA sequences in other *Brassica* species (*B. rapa*/*B. napus*/*B. oleracea*). Out of the 27 identical sequences 9, 26 and 3 sequences were present in *B. rapa*, *B. napus* and *B. oleracea*, respectively. An overlap of conserved miRNAs identified in *B. juncea* with previously reported miRNAs in *B. rapa*, *B. napus* and *B. oleracea* is shown in supplementary figure S3. In addition to conservation with other Brassica species, we also checked for the *B. juncea* conserved miRNAs that were previously reported in *A. thaliana*/*A. lyrata*. We found that 35 out of the 51 miRNAs were also reported previously in *Arabidopsis* (Table 3). A Venn diagram showing the number of common miRNAs in *B. juncea* and *Arabidopsis* species is provided in supplementary figure S4.

**Table 3:** The miRNA sequences identified in *B. juncea* were analysed for identical matches in family *Brassica*ceae.

*B. juncea* miRNA Family	Sequence	*B. rapa*	*B. napus*	*B. oleracea*	*A. thaliana*	*A. lyrata*
mir156	UGACAGAAGAGAGUGAGCAC	–	bna-miR156d/e/f	–	ath-miR156a/b/c/d/e/f	aly-miR156-5p-a/b/c/d/e/f
mir156	CGACAGAAGAGAGUGAGCAC		–	–	ath-miR156g	aly-miR156g-5p
mir157	UUGACAGAAGAUAGAGAGCAC	bra-miR157a	bna-miR156b/c/g	bol-miR157a	ath-miR157a/b/c	aly-miR157-5p-a/b/c
mir158	UCCCAAAUGUAGACAAAGCA	–	–	–	ath-miR158a	aly-miR158a-3p
mir159	UUUGGAUUGAAGGGAGCUCUA	bra-miR159a	bna-miR159	–	ath-miR159a	aly-miR159a-3p
mir160	UGCCUGGCUCCCUGUAUGCCA	bra-miR160a-5p	bna-miR160a/b/c/d	–	ath-miR160a/b/c	aly-miR160-5p-a/b/c
mir161	UCAAUGCACUGAAAGUGACUA	–	bna-miR161	–	–	–
mir164	UGGAGAAGCAGGGCACGUGCA	bra-miR164a	bna-miR164a	–	ath-miR164a/b	aly-miR164-5p-a/b
mir164	UGGAGAAGCAGGGCACGUGCG	–	bna-miR164b/c/d	–	ath-miR164c	aly-miR164c-5p
mir165	UCGGACCAGGCUUCAUCCCCC	–	bna-miR166f	–	ath-miR165a/b	–
mir166	UCGGACCAGGCUUCAUUCCCC	–	bna-miR166a/b/c/d/e	–	ath-miR166a/b/c//d/e/f/g	aly-miR166-3p-a/b/c/d/e/f/g/h
mir167	UGAAGCUGCCAGCAUGAUCUA	bra-miR167a/b/c/d	bna-miR167c	–	ath-miR167a/b	aly-miR167-5p-a/b
mir168	UCGCUUGGUGCAGGUCGGGAA	–	bna-miR168a	–	ath-miR168a/b	aly-miR168-5p-a/b
mir169	CAGCCAAGGAUGACUUGCCGG	–	bna-miR169n	–	ath-miR169b/c	aly-miR169-5p-b/c
mir169	UAGCCAAGGAUGACUUGCCUA	–	bna-miR169c/d/e/f	–	–	–
mir171	UGAUUGAGCCGCGCCAAUAUC	bra-miR171e	bna-miR171f	–	ath-miR171a	aly-miR171a-3p
mir171	AGAUAUUAGUGCGGUUCAAUC	–	–	–	–	aly-miR171b-5p
mir172	AGAAUCUUGAUGAUGCUGCAU	bra-miR172a/3p-b	bna-miR172a	bol-miR172a	ath-miR172a/3p-b	aly-miR172-3p-a/b
mir172	GGAAUCUUGAUGAUGCUGCAU	–	bna-miR172b/c	–	ath-miR172e	–
mir319	UUGGACUGAAGGGAGCUCCCU	–	–	–	ath-miR319a/b	aly-miR319-3p-a/b
mir319	UUGGACUGAAGGGAGCUCCUU	–	–	–	ath-miR319c	aly-miR319-3p-c/d
mir390	AAGCUCAGGAGGGAUAGCGCC	–	bna-miR390a/b/c	–	ath-miR390a	aly-miR390-5p-a/b
mir391	UUCGCAGGAGAGAUAGCGCCA	–	–	–	ath-miR391	aly-miR391-5p
mir393	UCCAAAGGGAUCGCAUUGAUCC	–	–	–	ath-miR393a	aly-miR393-5p-a/b
mir394	UUGGCAUUCUGUCCACCUCC	–	bna-miR394a/b	–	ath-miR394a/b	aly-miR394-5p-a/b
mir395	CUGAAGUGUUUGGGGGAACUC	–	bna-miR395a/b/c	–	ath-miR395a/d/e	aly-miR395-3p-d/e/g
mir395	CUGAAGUGUUUGGGGGGACUC	–	bna-miR395d/e/f	–	ath-miR395b/c/f	aly-miR395-3p-b/f/h
mir395	CUGAAGUGUUUGGAGGAACUC	–	–	–	–	aly-miR395i
mir396	GCUCAAGAAAGCUGUGGGAAA	–	–	–	–	aly-miR396b-3p
mir397	UCAUUGAGUGCAGCGUUGAUGU	–	bna-miR397a/b	–	–	–
mir398	GGAGUGUCAUGAGAACACGGA	–	–	bol-miR398a-5p	–	–
mir399	UGCCAAAGGAGAGUUGCCCUG	–	–	–	ath-miR399b/c	aly-miR399-3p-b/c
mir399	UGCCAAAGGAGAUUUGUCCGG	–	bna-miR399c	–	–	–
mir399	UGCCAAAGGAGAUUUGCCCGG	–	bna-miR399a/b	–	ath-miR399f	aly-miR399-3p-a/h, aly-miR399j
mir828	UCUUGCUUAAAUGAGUAUUCCA	–	–	–	ath-miR828	aly-miR828-5p
mir845	CGGCUCUGAUACCAAUUGAUG	–	–	–	ath-miR845a	–
mir858	UUCGUUGUCUGUUCGACCUUG	–	–	–	ath-miR858b	–
mir2111	GUCCUCGGGAUGCGGAUUACC	bra-miR2111a-3p	bna-miR2111a-3p	–	ath-miR2111a-3p	aly-miR2111a-3p
mir2111	AUCCUCGGGAUACAGAUUACC	–	bna-miR2111b-3p	–	–	aly-miR2111b-3p
mir2111	UAAUCUGCAUCCUGAGGUUUA	bra-miR2111-5p-a/b	bna-miR2111d/5p-b/d	–	ath-miR2111-5p-a/b	aly-miR2111-5p-a/b

The miRNA family, sequence and miRBase nomenclature of these identical miRNA sequences of each species is presented.

The miRNA sequences that remained after exclusion of conserved and their variants were considered as novel. These sequences exhibited a minimum of three or more mismatches with the conserved miRNAs. We were able to discover 126 novel miRNAs many of which were represented in multiple conditions. The novel miRNAs has been named as Bju-Nx (for *Brassica juncea* Novel, x being the number) hereafter in the text. We were also able to sequence star sequences for 37 of 126 novel miRNAs. The miRNAs whose star sequence deduced from the precursor was also present in the sequenced datasets were termed as “true novel” miRNAs, whereas the miRNAs whose star miRNA sequences were not present in the sequenced datasets were termed as “candidate novel” miRNAs. Additional details on the sequence, read counts and precursors of “true novel” miRNAs are presented in supplementary tables S6 and S7. Similar information for the “candidate novel” miRNAs is presented in supplementary tables S8 and S9. For many of the true novel and candidate novel miRNAs more than one precursor was identified (Supplementary Table S7 and S8). Fold changes derived from normalized expression values revealed that many of the identified novel miRNAs have atleast two fold up/down modulation in their expression as compared with control. Salinity stress influenced expression of maximum number of miRNAs (96) followed by high temperature stress (45) and drought stress (37). Many of these miRNAs were regulated by more than one stress condition. For example: 23 miRNAs were regulated by salinity and high temperature, 17 miRNAs being regulated by both salinity and drought stress and six miRNAs were regulated by both drought and high temperature stress. Interestingly, expression of 10 miRNAs were influenced by all the three stresses. A Venn diagram representing the novel miRNAs that are regulated by abiotic stresses is presented in supplementary figure S2.

**Figure 1:**
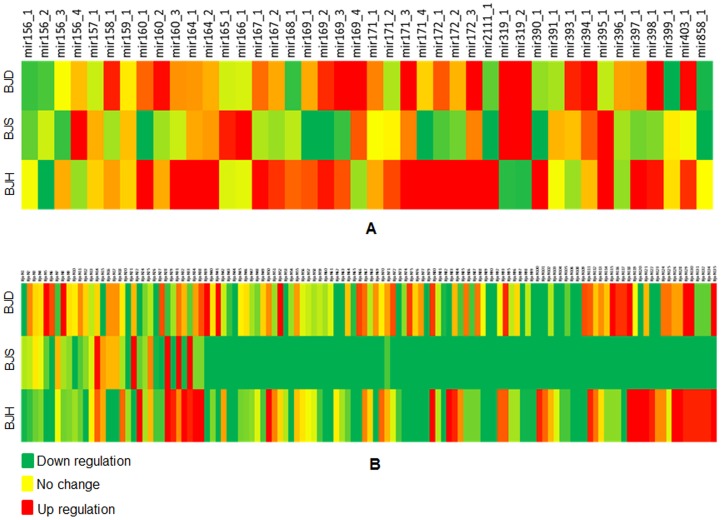
The digital expression values obtained through high-throughput sequencing were normalized using TPM (transcripts per million) method and used to calculate digital expression fold change [Log 2 (abiotic stress treated sample/control sample)] in high temperature treated (BJH), salinity stress treated (BJS) and drought stress treated (BJD) *B. juncea* seedlings library with respect to untreated seedlings library. Thus deduced digital expression fold change is presented as heat map for conserved (A) and novel (B) miRNA sequences.

Both the conserved and the novel miRNAs had varied expression. The TPM (Transcripts Per Million) of the highest expressing conserved miRNAs was enormously different than that of the highest expressing novel miRNAs. For the conserved miRNAs, miR156_1 was the most abundant molecule with a read count of 3,66,434 in control sample. Among the 37 true novel miRNAs (miRNAs whose star sequences were also discovered), Bju-N1 was the most abundant with expression count of 6575 followed by Bju-N2 and Bju-N3 each having expression count of 3340 and 388, respectively, in the control sample. The minimum read count was 1 for Bju-N36 and Bju-N37. Nineteen of the true novel miRNAs had expression less than 10 reads, 13 true novel miRNAs had expression between 10–100, and 5 true novel miRNAs had expression more than 100 reads. In case of candidate novel miRNAs (whose star sequence were not discovered), Bju-N38 (249 read counts) was moderately abundant followed by Bju-N39 (155 read counts) and Bju-40 (146 read counts). Bju-N135 and Bju-N136 were the least abundant candidate miRNAs with each having 2 read counts. Fifty eight miRNAs in the candidate novel miRNA dataset had less than 10 reads, 38 miRNAs had expression between 10–100 while only 3 had read count above 100. A heat map representing fold change deduced from the normalized digital expression for the conserved miRNAs, true novel miRNAs and candidate novel miRNAs is represented in Figure 1.

### Expression Analysis of the miRNAs

**Figure 2:**
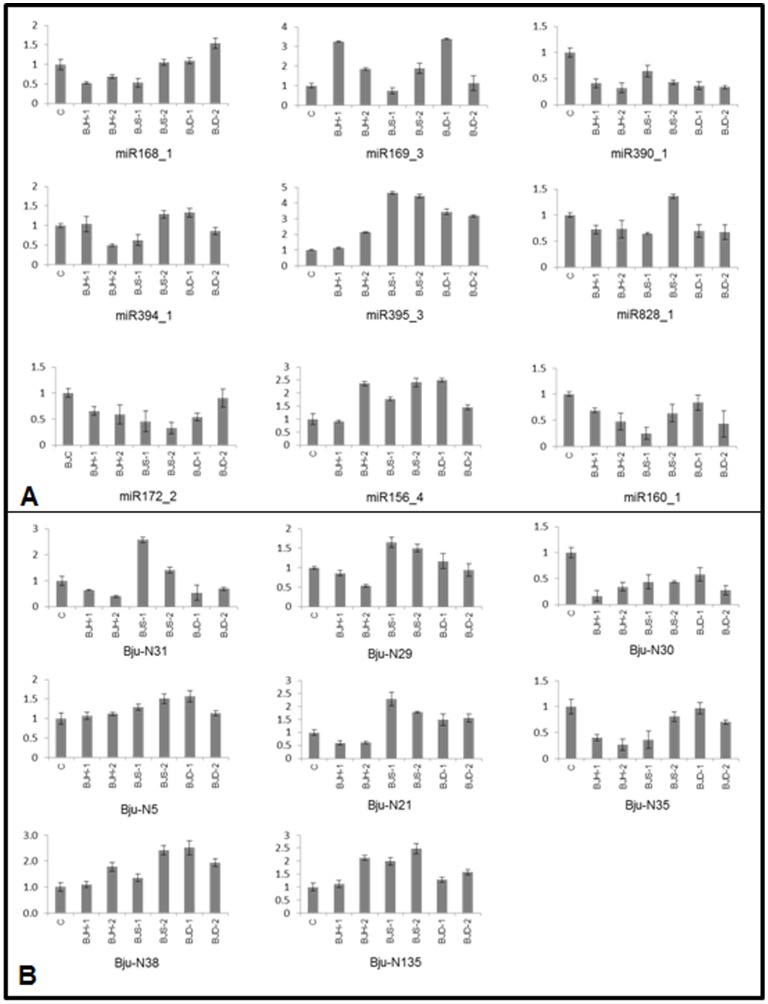
Expression profiling of conserved (A) and novel miRNAs (B) was performed to validate the predicted miRNAs. Quantitative PCR was performed using TaqMan chemistry. The relative abundance (Y-axis) was calculated using the ΔΔCt method. *B. juncea* seedlings were subjected for varied durations to either high temperature stress either at 35°C (BJH-1) and 42°C (BJH-2) or salinity stress at 150 mM NaCl (BJS-1) and 250 mM NaCl (BJS-2) or drought stress using 20% PEG (BJD-1) and 300 mM mannitol (BJD-2). The mean of three independent biological replicates is presented.

A few of the conserved, true novel and candidate novel miRNAs were selected for experimental confirmation and relative abundance measurement across different stresses. TaqMan based quantitative real time PCR approach was utilized for this examination. Expression of all the selected miRNAs was easily detectable by QPCR. Out of the six conserved miRNAs tested (miR168_1, miR169_3, miR172_2, miR390_1, miR394_1, miR395_2 and miR828_1) expression of miR395_2 showed significant upregulation in all the stresses while miR390_1 and miR172_2 was found downregulated in all the stresses. Similarly out of the six true novel miRNAs (Bju-N31, Bju-N29, Bju-N30, Bju-N21, Bju-N5 and Bju-N35), Bju-N30 was down regulated in all the stresses. Two miRNAs, Bju-N29 and Bju-N21, were specifically upregulated in salt stress and drought stress while down regulated in high temperature stress. Bju-N31 showed high accumulation under salinity stress. Expression of Bju-N35 was repressed in high temperature and salinity stress while no significant change was observed in drought stress. In addition to true novel miRNAs, expression for two of the candidate novel miRNAs namely Bju-N38 and Bju-N135 was also determined. Both miRNAs (Bju-N38, Bju-N135) showed significant upregulation in abiotic stress conditions. The relative abundance profiles as measured by real time PCR are depicted in Figure 2.

### Target Prediction of Conserved and Novel miRNAs

A single miRNA is known to regulate expression of many genes or members of a gene family. To find out the putative targets of both the conserved and novel miRNAs, we used open source software “plant small RNA target”. As transcript datasets for *B. juncea* are not yet available, we predicted the targets on the basis of *B. rapa* dataset. Most of the miRNAs discovered in this study were found to have multiple hits (see Supplementary Table S10 for targets of conserved miRNA and S11 for targets of novel miRNAs). For example, among the conserved miRNAs, 19 miRNAs have ≥10 hits while the number of targets predicted for 23 of the conserved miRNAs ranged between 2–9. Only one target was predicted for four miRNAs, five miRNAs did not receive any hit. A total of, 379 targets were identified for 46 conserved miRNAs. Similarly, novel miRNAs were also predicted to target multiple genes. No targets were predicted for 24 novel miRNAs. For the remaining 92 novel miRNAs a total of 450 targets were predicted. Bju-N3 received 23 target hits followed by Bju-N48 and Bju-N66 which received 22 and 16 target hits, respectively. Nine novel miRNAs had 10 or more number of predicted targets and for 71 novel miRNAs the number of targets ranged between 2–9. Twenty novel miRNAs were predicted to have single targets. The predicted targets of novel miRNAs profiled by qPCR (Bju-N31, Bju-N29, Bju-N30, Bju-N21, Bju-N35, Bju-N38, Bju-N135, Bju-N5) in different abiotic stresses are listed in Table 4. The targets of these novel miRNAs are involved in variety of functions including calcium signaling, ABA catabolism and biotic defense responses.

**Table 4:** Putative targets of selected novel miRNAs.

miRNA	Target accession	Target description
Bju-N21	Bra019452	MAK10-like protein
	Bra031115	Abnormal Leaf Shape 2 (ALE2)
	Bra011405	Glycine decarboxylase P-protein 1 (AtGLDP1)
Bju-N29	Bra030020	ECA1 gametogenesis related family protein
Bju-N30	Bra036733	StaR-like protein domain-containing protein
	Bra005780	Exostosin family protein
	Bra019452	MAK10-like protein
	Bra016455	Calcium-binding EF hand-containing protein
Bju-N31	Bra027793	Signal peptide peptidase-like 2
Bju-N35	Bra003401	Beta glucosidase 16-like
	Bra011494	D111/G-patch domain-containing protein
	Bra011491	Uncharacterized protein
	Bra006393	Tetratricopeptide repeat-containing protein
	Bra024652	TIR-NBS-LRR class disease resistance protein
Bju-N38	Bra037341	Cryptic Precocious (CRP)
	Bra023716	Structural Maintenance of Chromosomes 6B (SMC6B)
Bju-N135	Bra021844	MOS4-ASSOCIATED COMPLEX 3B (MAC3)
Bju-N40	No target found	–

Expression levels of few identified novel miRNAs was determined using quantitative PCR. The putative targets of these novel miRNAs were predicted in *B. rapa* using psRNAtarget software. The accession number and description of predicted targets (based on their homology with *A. thaliana*) for each novel miRNA are presented.

**Figure 3:**
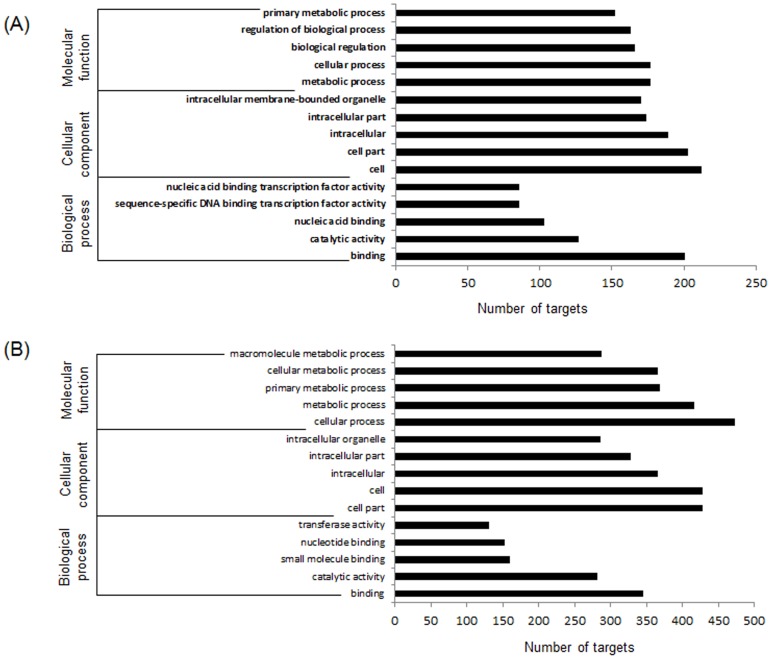
Gene ontology analysis of predicted targets of conserved (A) and novel (B) miRNAs. For each GO category (molecular function, cellular component and biological process) top 5 GO terms obtained through Blast2GO resource (www.blast2go.com) are presented.

Predicted targets of conserved and novel miRNAs were subjected to gene ontology (GO) analysis. It was observed that majority of the targets of conserved and novel miRNAs were involved in various cellular (177 targets of conserved miRNAs and 473 targets of novel miRNAs) and metabolic (177 targets of conserved miRNAs and 416 targets of novel miRNAs) processes. Among the conserved miRNA targets, 201 targets had binding ability, 127 targets had catalytic activity, 103 targets had nucleic acid binding activity and 86 targets had sequence-specific DNA binding/transcription factor activity. Similarly in case of predicted targets of novel miRNAs, 345 targets had binding property, 281 targets had catalytic activity, 160 targets had small molecule binding property, 153 targets had nucleotide binding activity and 130 targets had transferase activity. Most of the targets of both conserved and novel miRNAs were linked to cell (212 targets of conserved miRNAs and 428 targets of novel miRNAs) and cell part (203 targets of conserved miRNAs and 428 targets of novel miRNAs). The frequency of various GO terms associated with predicted targets of conserved and novel miRNAs have been depicted in Figure 3.

### Experimental Validation of Predicted Targets and Expression Profiling

**Figure 4:**
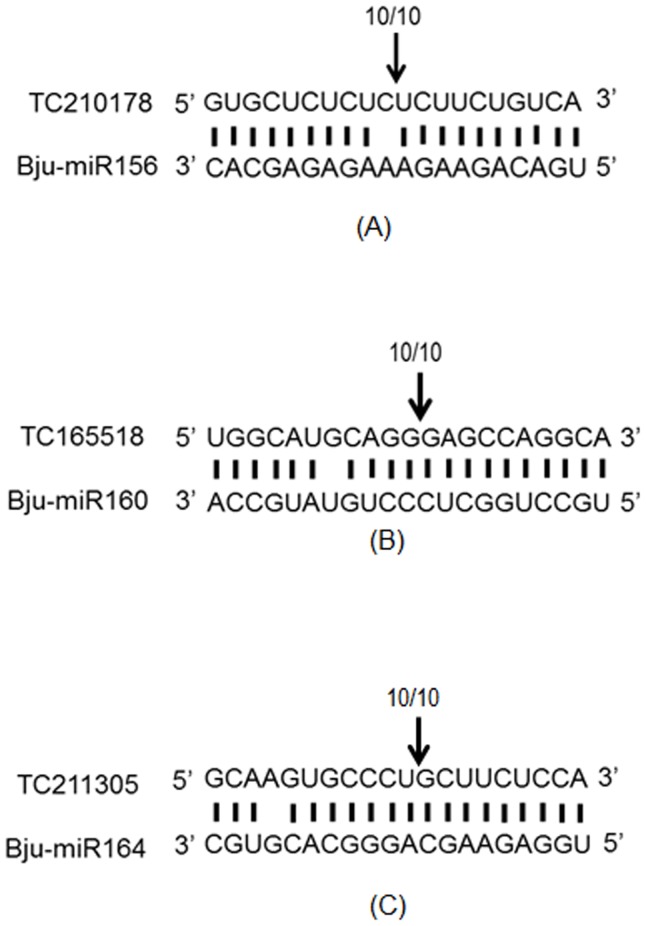
Cleavage site mapping of selected miRNAs using modified RLM-RACE. Targets of the *B. juncea* miR156 (Bju-miR156), miR160 (Bju-miR160) and miR164 (Bju-miR164) were predicted by psRNA target finder. Primers were designed on the basis of the predicted sequences (TC2101178, TC165518 and TC211305). Cleavage products of three miRNA targets were amplified from RACE library, cloned in pGEMT vector and sequenced. The binding region between the miRNAs and the targets is shown. The arrows indicate cleavage site of transcript and the number of positive clones out of the total clones sequenced is indicated in parentheses above the arrow.

Based on the previous reports targets of a few conserved miRNAs were shortlisted for further validation using a modified RNA ligase mediated random amplification of cDNA ends (RLM-RACE). As the *B. rapa* dataset was not available until recently, the information of the primers used for RLM-RACE was derived from *B. napus*. The RLM-RACE was carried out for *B. juncea* miRNAs miR156_4, miR160_1 and miR164_1. The target for Bju-miR156_4 (TC210178) is a homologue of *A. thaliana* SPL2 (Squamosa Promoter binding Like-2). All clones of SPL2 were found to be cleaved between 10–11 bases from the 5′ end of the miRNA: target hybrid (Figure 4A). Similarly, the target for Bju-miR160_1 (TC165518) is a homologue of *A. thaliana* ARF17 (Auxin Response Factor 17).

**Figure 5:**
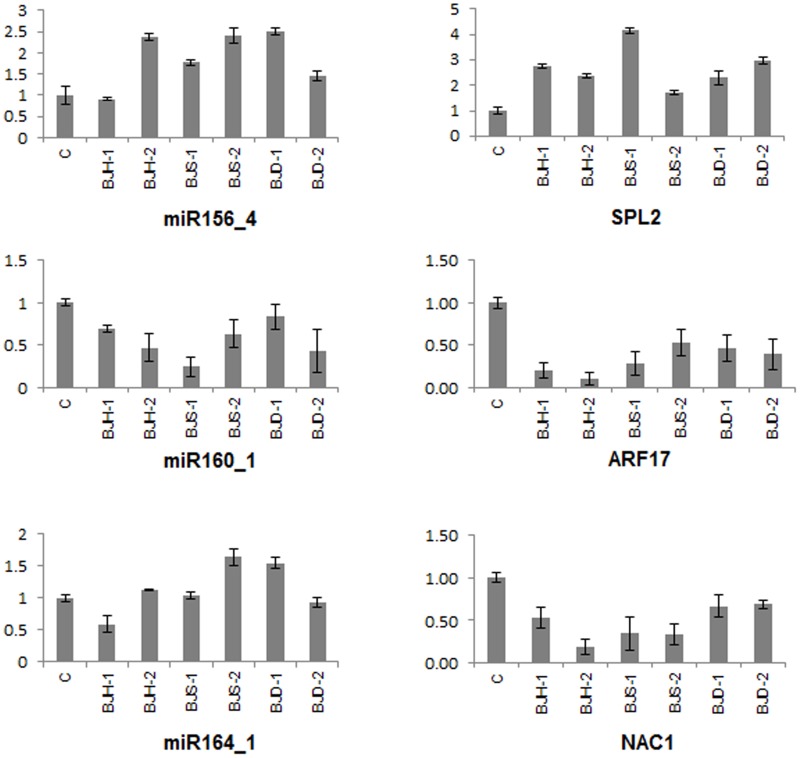
Expression profiling of miRNAs and their targets. The relative abundances of miR156, miR160, miR164 and their respective targets i.e., SPL2 (TC2101178), ARF17 (TC165518) and NAC1 (TC211305) was measured under different abiotic stress conditions using quantitative real time PCR. *B. juncea* seedlings were subjected for varied durations to either high temperature stress either at 35°C (BJH-1) and 42°C (BJH-2) or salinity stress at 150 mM NaCl (BJS-1) and 250 mM NaCl (BJS-2) or drought stress using 20% PEG (BJD-1) or 300 mM mannitol (BJD-2). The mean of three independent biological replicates is presented.

Similar to cleavage of TC210178, all the clones of ARF17 homologue (TC165518) had a cleavage site between 10–11 bases from the 5′ end of the miRNA: target hybrid (Figure 4B). In case of TC211305 (target of miR164_1 and a homolugue of *A. thaliana* NAC1) all clones had a cleavage site between 9–10 bases from 5′ end of the miRNA binding site (Figure 4C). The sequence and additional details of these validated miRNA targets are provided in supplementary table S12. Expression analysis of miR156_4, miR160_1, miR164_1 and their respective targets (SPL2, ARF17 and NAC1) was also carried out using real time PCR. A comparison of expression profiles of these miRNAs and their respective targets under different abiotic stresses is depicted in Figure 5. miR156_4 and miR164_1 were upregulated while miR160_1 was downregulated in all the stresses. On the other hand, SPL2-like (TC210178) was upregulated while ARF17-like (TC165518) and NAC1-like (TC211305) were downregulated in all the stresses.

## Discussion

We constructed small RNA libraries from untreated and abiotic stress treated *B. juncea* seedlings. Highthroughput sequencing yielded approximately 25 million reads which were subsequently processed to remove inherent redundancy. Finally, 12.6 million unique sequences were obtained. A very low percentage of the unique sequences were derived from degraded fragments of tRNA, rRNA and sn/snoRNA, which is suggestive of high-quality RNA employed for library construction. Majority of the reads were distributed in a narrow size range of 20–24 nucleotides thereby implicating the role of Dicer proteins in generation of these molecules. The highest proportion of the sequenced RNAs was of 24 nt in putative small RNA data. This result is in agreement with several other studies wherein 24 nt long sRNAs constituted the majority of small RNA population [67]–[71]. Based on the 100% homology with miRBase sequences, 51 conserved miRNAs belonging to 30 families were identified in *B. juncea*. However, due to lack of genomic sequence of *B. juncea,* the complete repertoire of conserved miRNAs could not be discovered. A well annotated genome draft would also make it possible to differentiate identical mature miRNA sequences mapped at different genomic loci, thus increasing the number of miRNA family members. A large portion of sequenced data remained unmapped and is a potential source for discovery of additional miRNAs along with their precursor sequences on availability of draft genome sequence of *B. juncea*. Previously, 43, 54 and 7 conserved miRNA sequences have been reported from *B. napus*, *B. rapa* and *B. oleracea*, respectively. Collectively these miRNAs represent 79 unique sequences. Out of the 79 miRNAs, we were able to identify 27 identical sequences. Additionally, 24 conserved miRNAs not reported so far in any *Brassica* species were also discovered.

We identified 126 novel miRNAs in our dataset. The expression of *B. juncea* miRNAs has been previously studied by two independent groups. In the first study expression of only the conserved microRNAs was studied using microarray [72]. A more comprehensive study using high-throughput sequencing was carried out by Yang *et al.* (2013), resulting in discovery of a sizeable population of conserved and novel miRNAs [47]. However, Yang *et al.* used *Arabidopsis* genomic resources for mapping and identification of precursor sequences of miRNAs while we have generated and utilized specifically *B. juncea* genomic reference in addition to the other publically available *Brassica* resources as reference for small RNA mapping and precursor identification.

We discovered 32 and 37 star miRNA sequences for the 51 conserved and 126 novel miRNAs, respectively. Several studies have demonstrated that invariably a higher number of mature miRNA are discovered than the star miRNAs because during miRNA biogenesis, star sequence is rapidly degraded [12], [73]–[77]. Our analysis reveals that higher expression levels of mature sequence cannot be correlated with the discovery of the counterpart star sequence as we have identified star sequences for even the less abundant mature miRNAs (frequency as low as 1 TPM). Apparently stability of the star miRNA determines its steady state levels and therefore a greater depth sequencing coverage could possibly reveal additional star miRNA sequences. It will be worthwhile to study the biological significance of star miRNAs in *B. juncea* as previous studies have indicated that individual strands of miRNA duplex are used as guide strands to regulate genes involved in distinct processes [78], [79].

We used publicly available genomic and transcriptomic datasets from different *Brassica* species for mapping of the small RNA sequences. Additionally, we also generated a reference genome of *B. juncea* at 8× coverage using paired end sequencing on Illumina GAIIx onto which the small RNA sequences were mapped. The largest percentage (28.6%) of putative small RNA population mapped to *B. juncea* dataset followed by *B. rapa* genome dataset (18%; Supplementary Table S13). However the number of precursors that folded into canonical hairpin structure was higher in sequences extracted from *B. rapa* than *B. juncea* resources. We believe that this is essentially due to the partially assembled contigs of the *B. juncea* dataset. Conceivably, additional conserved and novel miRNAs can be identified from a better assembled genomic framework in *B. juncea*.

Digital gene expression of predicted miRNAs shows that majority of miRNAs were expressed in all three stresses included in this study. However, the expression of many of these varied among different stresses. Previous studies have also demonstrated stress specific regulation of miRNAs [24], [29], [80]–[83]. Based on the resources available in *B. napus* and *B. rapa*, we predicted the targets of conserved and novel miRNAs using psRNA target finder. Multiple targets were predicted for many miRNAs as has been reported previously [84], [85]. The exact role of miRNAs and their targets in controlling abiotic stress responses in *B. juncea* will be evident with gain/loss of function studies. GO analysis of the targets predicted for the novel miRNAs indicate, that a substantial number of them have nucleic acid binding property and may therefore represent transcription factors.

Two approaches were chosen to validate the targets of conserved miRNAs. Expression profiling of miRNA and its predicted target was carried out for ascertaining inverse correlation and RLM-RACE was exploited to map the cleavage site in the target. A coordinated interplay between miR156 and miR172 levels mediated by SPL2 (target of miR156) determines the phase changes in plants [86]–[88]. Increased levels of miR156 are concomitant with decreased levels of SPL2 and miR172 during developmental transition [89], [90]. Our expression data reveals an inverse concurrence between the levels of miR156 and miR172 in abiotic stresses. Moreover RLM-RACE results demonstrate that SPL2 transcript is cleaved *in vivo*. However, an inverse corelation in the levels of miR156 and SPL2 (amplified on the basis of information available in *B. napus*) was not observed in the present study. SPL family is represented by 17 members in *Arabidopsis* out of which 11 are targeted by miR156 family members. In light of the polyploid and complex genome it is conceivable that many more SPL members are present in *B. juncea*, and SPL2 might not be an authentic target of miR156 under conditions of abiotic stress. In future, *de novo* transcriptome and degradome sequencing under these conditions can be exploited to reveal the true targets for miR156. Nevertheless, inverse correlation between the miR156 and miR172 levels under abiotic stress conditions and presence of an *in vivo* cleaved SPL2 mRNA, expands the prospects of miR156-SPL-miR172 cascade playing an imperative role in response to abiotic stresses. Similar to the pair of miR156 and its predicted target SPL2, we observed that mRNA of ARF17 (a predicted target for miR160) was cleaved *in vivo* between 10–11 position from 5′ end of miR160 binding site. However transcripts level of ARF17 did not exhibit an inverse correlation with the miR160 levels. In contrast NAC1 (a predicted target for miR164) displayed an inverse correlation with miR164 levels. Both ARF and NAC proteins are involved in auxin homeostasis and may therefore regulate stress tolerance through a complex interaction of plant hormones.

Expression of a few novel miRNAs was also detected by QPCR and the results indicate that some of these miRNAs might be involved dynamically involved in modulating expression of stress related factors. Of particular interest are predicted targets of BjuN21 and BjuN35. One of the targets that can possibly be targeted by BjuN21 is glycine decarboxylase (GDC) P-protein. This protein is a part of glycine cleavage complex which in conjugation with serine hydroxymethyltransferase aids in conversion of glycine to serine. Partial involvement of glycine decraboxylase in beatine accumulation has been indicated and therefore regulation of glycine decraboxylase by BjuN21 can possibly moderate the levels of osmoprotectant glycine betaine [91]. Additionally, GDC inhibition is sufficient to mount an oxidative burst which results in programme cell death [92]. Programme cell death is a defence mechanism elicited by plants in response to invading pathogens. In this context it will be worthwhile to investigate the expression of BjuN21 under biotic stresses in *B. juncea*. BjuN35 potentially targets β-glucosidase 16 like gene. There are 48 members of β-glucosidase gene family in *Arabidopsis* and there is irrefutable evidence implicating some of them in modulating cellular ABA levels. Oxidation as well as conjugation plays an important role in catabolism of ABA. Conjugation of glucose with ABA renders it inactive and it has been shown that β-glucosidase removes glucose from ABA in response to dehydration stress thereby rapidly replenishing the cellular ABA pool [93]. Additionally, over-expression of *A. thaliana* β-glucosidase gene has been shown to increase drought resistance in *Arabidopsis* and creeping bentgrass [94], [95]. It is conceivable that BjuN35 regulates active ABA levels in *B. juncea* by managing levels of β-glucosidase transcripts.

In conclusion, we have generated in this study, the first comprehensive abiotic stress influenced small RNA dataset in *B. juncea*. The combinatorial approach of NGS and computational methods led to the discovery of 51 conserved and 126 novel miRNAs. The present study provides a holistic view of *B. juncea* miRNAome under conditions of high temperature, salinity and drought. The catalogue of miRNA sequences, their expression and putative targets, generated in this study can be utilized to design crop improvement strategies in *B. juncea* and related species.

## Supporting Information

Figure S1 **Size distribution of the putative small RNA population.** Following elimination pipeline both the redundant (represented by striped bars) and the unique (represented by black bars) small RNAs were analyzed for their length (X-axis) versus their absolute number (Y-axis). Most of the small RNAs are in the size range of 20–24 nucleotides.
(XLSX)

Figure S2 **Digital gene expression was used to categorize both (A) conserved and (B) novel miRNAs that were responsive to high temperature (BJH), salinity (BJS) and drought stress (BJD).** The number of miRNAs that were regulated exclusively or by more than one stress condition has been presented.
(XLSX)

Figure S3 **Overlap of conserved miRNAs identified in** ***B. juncea*** **with previously reported miRNAs in** ***B. rapa, B. napus and B. oleracea*** **.**
(XLSX)

Figure S4 **Overlap of conserved miRNAs identified in** ***B. juncea*** **with previously reported miRNAs in** ***A. thaliana*** **and** ***A. lyrata*** **.**
(XLSX)

Table S1 **Details of primers used in this study.**
(XLSX)

Table S2 **The total number of purity filtered sRNA reads obtained through high-throughput sequencing and the number of sRNA reads having adapter sequence both in redundant and unique datasets.**
(XLSX)

Table S3 **Conserved miRNA sequences identified in** ***B. juncea*** **with their absolute counts, normalized counts and fold change values in control and different abiotic stress conditions.**
(XLSX)

Table S4 **Precursor sequences of conserved miRNAs.**
(XLSX)

Table S5 **Variants of conserved miRNA sequences identified in** ***B. juncea.***
(XLSX)

Table S6 **True novel miRNA sequences identified in** ***B. juncea*** **with their absolute counts, normalized counts and fold change values in control and different abiotic stress conditions.**
(XLSX)

Table S7 **Precursor sequences of true novel miRNAs.**
(XLSX)

Table S8 **Candidate novel miRNA sequences identified in** ***B. juncea*** **with their absolute counts, normalized counts and fold change values in control and different abiotic stress conditions.**
(XLSX)

Table S9 **Precursor sequences of candidate novel miRNAs.**
(XLSX)

Table S10 **Putative targets of conserved miRNAs predicted in** ***B. rapa*** **.**
(XLSX)

Table S11 **Putative targets of true novel and candidate novel miRNAs predicted in** ***B. rapa*** **.**
(XLSX)

Table S12 **Sequences and additional details of targets validated by 5′ RLM-RACE.**
(XLSX)

Table S13 **Mapping summary of putative small RNA population.**
(XLSX)
