# Correction: A Genome-Wide Perspective of miRNAome in Response to High Temperature, Salinity and Drought Stresses in *Brassica juncea* (Czern) L

**DOI:** 10.1371/journal.pone.0125644

**Published:** 2015-04-13

**Authors:** The PLOS ONE Staff

S3 Table is a duplicate of S13 Table. Please view the correct S3 Table below.

## Supporting Information

S3 Table Conserved miRNA sequences identified in *B*. *juncea* with their absolute counts, normalized counts and fold change values in control and different abiotic stress conditions. (XLSX)
